# Central adiposity in relation to risk of liver cancer in Chinese adults: A prospective study of 0.5 million people

**DOI:** 10.1002/ijc.32148

**Published:** 2019-02-13

**Authors:** Yuanjie Pang, Christiana Kartsonaki, Yu Guo, Yiping Chen, Ling Yang, Zheng Bian, Fiona Bragg, Iona Y. Millwood, Canqing Yu, Jun Lv, Junshi Chen, Liming Li, Michael V. Holmes, Zhengming Chen

**Affiliations:** ^1^ Clinical Trial Service Unit and Epidemiological Studies Unit (CTSU) Nuffield Department of Population Health, University of Oxford Oxford United Kingdom; ^2^ Medical Research Council Population Health Research Unit (MRC PHRU), Nuffield Department of Population Health University of Oxford Oxford United Kingdom; ^3^ Chinese Academy of Medical Sciences Beijing China; ^4^ School of Public Health Peking University Beijing China; ^5^ National Center for Food Safety Risk Assessment Beijing China; ^6^ National Institute for Health Research Oxford Biomedical Research Centre Oxford University Hospital Old Road, Oxford United Kingdom

**Keywords:** central adiposity, liver cancer, Chinese, cohort study

## Abstract

Central adiposity is associated with liver cancer risk beyond general adiposity in Western populations. However, there is little prospective evidence in East Asian populations who are more likely to have central adiposity at given BMI levels. The prospective China Kadoorie Biobank recruited 512,713 adults aged 30–79 years from 10 diverse areas. During 10 years follow‐up, 2,847 incident cases of liver cancer were identified. Cox regression was used to estimate adjusted hazard ratios (HR) for liver cancer associated with central adiposity, excluding individuals with cancers and liver diseases at baseline and the first 5 years of follow‐up (1,049 incident liver cancer cases). Overall, mean waist circumference (WC) was 82.2 (SD 9.8) cm in men and 79.1 (9.5) cm in women. Central adiposity showed positive associations with liver cancer risk. Associations were strongest for WC and waist‐to‐hip ratio (WHR), with adjusted HRs per 1‐SD of 1.09 (95%CI 1.01–1.18) and 1.12 (1.02–1.23), respectively. The positive associations became stronger when additionally adjusting for BMI (1.26 [1.09–1.46] and 1.14 [1.02–1.28]). The positive association of central obesity (WC ≥90 cm in men and ≥ 80 cm in women) with liver cancer increased progressively with the number of other presenting metabolic risk factors (physical inactivity, diabetes, and hypertension), with HRs of 1.07 (0.90–1.28), 1.17 (1.00–1.38), and 1.91 (1.40–2.59) in those with one, two, and three factors (*p* for trend 0.006). In this relatively lean Chinese population, there were positive associations of central adiposity with risk of liver cancer, with WHR and WC showing the strongest associations.

## Abstract 2

**What's new?**

In this relatively lean Chinese population, central adiposity was positively associated with risk of liver cancer, and the positive association was stronger at given BMI levels. Among central adiposity traits examined, waist circumference and waist‐to‐hip ratio were the best indicators of liver cancer risk. The association of central adiposity with liver cancer risk increased progressively with the number of other presenting metabolic risk factors, including physical inactivity, diabetes, and hypertension.

**Abbreviations**
%BF: percent body fat
BMI: body mass index
CI: confidence interval
CKB: China Kadoorie Biobank
CVD: cardiovascular disease
EPIC: European Prospective Investigation into Cancer and Nutrition
HBsAg: hepatitis B surface antigen
HBV: hepatitis B virus
HC: hip circumference
HR: hazard ratio
ICD‐10: International Classification of Diseases, 10th Revision
IDF: International Diabetes Federation
MET: metabolic equivalent of task
NAFLD: non‐alcoholic fatty liver disease
SBP: systolic blood pressure
SD: standard deviation
WC: waist circumference
WHO: World Health Organisation
WHR: waist‐to‐hip ratio
WHtR: waist‐to‐height ratio

Prospective studies in Western populations have shown that measures of general (i.e. body mass index [BMI]) and central adiposity (i.e. waist circumference [WC] and waist‐to‐hip ratio [WHR]) are each associated with higher risk of liver cancer.^1^, ^2^, ^3^, ^4^ When examining the associations of these adiposity traits, previous studies in Western populations have suggested that central adiposity is associated with liver cancer risk beyond the risk conferred by general adiposity.^4^ Uncertainty remains, however, about which measure of central adiposity is the best indicator of liver cancer risk. Furthermore, there is limited evidence in East Asian populations on central adiposity and liver cancer risk. Understanding the associations between central adiposity and liver cancer risk is important because East Asians are more likely to have central adiposity than their Western counterparts at given BMI levels.^5^ Central adiposity is strongly associated with risk of non‐alcoholic fatty liver disease (NAFLD, a precursor on the pathological pathway to liver cancer) beyond BMI.^6^, ^7^ Although hepatitis B viral infection is the major risk factor for liver cancer in East Asia, NAFLD has emerged as an important risk factor due to rapid changes in lifestyle, such as physical inactivity and dietary patterns (i.e. increased energy intake).^7^, ^8^, ^9^ Therefore, we examined the associations of central adiposity with risk of liver cancer in the China Kadoorie Biobank (CKB). We also compared measures of central adiposity in predicting risk of liver cancer.

## Materials and Methods

### Study population

Details of the CKB design, survey methods, and population characteristics have been described elsewhere.^10^ Briefly, 512,713 participants aged 30–79 were recruited into the study from 10 (5 urban, 5 rural) localities in China between June 2004 and July 2008. The study areas were selected to provide diversity in risk factor exposure and disease patterns, while taking into account population stability, quality of mortality and morbidity registries, capacity, and long‐term commitment within the areas. All participants provided written informed consent. Prior international, national, and regional ethical approvals were obtained.

At local study assessment clinics, participants completed an interviewer‐administered laptop‐based questionnaire on socio‐demographic characteristics, smoking, alcohol consumption, diet, physical activity, personal and family medical history, and current medication. A range of physical measurements were recorded by trained technicians, including height, weight, hip circumference (HC), WC, bioimpedance, lung function, blood pressure, and heart rate, using calibrated instruments with standard protocols. In addition, desktop analysers were used to measure random plasma glucose (Johnson & Johnson SureStep Plus Meter) and hepatitis B surface antigen (HBsAg) (ACON Biotech). A more detailed description of the methods and data source is presented in eMethods.

Central adiposity measures assessed include WC, waist‐to‐hip ratio (WHR), HC, and waist‐to‐height ratio (WHtR). General adiposity measures assessed include BMI, percent body fat (%BF), and weight. All anthropometric measurements were taken by trained technicians while participants were wearing light clothes and no shoes, usually to the nearest 0.1 cm or 0.1 kg. Standing height was measured using a stadiometer. Weight was measured using a body composition analyser (TANITA‐TBF‐300GS; Tanita Corporation), with subtraction of weight of clothing according to season (ranging from 0.5 kg in summer to 2.0–2.5 kg in winter). WC and HC were measured using a soft non‐stretchable tape. WC was measured at the midpoint between the lowest rib margin and the iliac crest. HC was measured at the maximum circumference around the buttocks. WHR was the ratio of WC to HC. WHtR was the ratio of WC to standing height. BMI was calculated as the measured weight in kilogrammes divided by the square of the measured height in metres. %BF was the fraction of total weight that was estimated to be fat weight by the Tanita body composition analyser using proprietary algorithms.

### Follow‐up for mortality and morbidity

The vital status of each participant was determined periodically through China Centre for Disease Prevention and Control's Disease Surveillance Points system,^11^ supplemented by regular checks against local residential records and health insurance records and by annual active confirmation through street committees or village administrators. Additional information about major diseases and any episodes of hospitalisation was collected through linkages, *via* each participant's unique national identification number, with disease registries (for cancer, ischaemic heart disease, stroke, and diabetes) and national health insurance claims databases, which have almost universal coverage in the study areas. All events were coded using International Classification of Diseases, 10th Revision (ICD‐10) by trained staff who were blinded to baseline information. By 1.1.2017, 42,921 (8%) participants had died and 5,276 (1%) were lost to follow‐up.

### Statistical analysis

Our study excluded individuals with a prior history of cancer (n = 2,577), cirrhosis or hepatitis (n = 6,321), or a positive HBsAg test (n = 24,900). To limit bias due to effects of pre‐existing diseases on baseline adiposity (i.e. reverse causality), we further excluded the first 5 years of follow‐up. After these exclusions, 478,042 individuals remained in the main analysis.

The mean values and prevalence of baseline characteristics were calculated by WC category, adjusted for age (in 5‐year groups), sex, and region using direct standardisation. Incidence rates of liver cancer were calculated using direct standardisation to the age, sex, and region structure of the population.

Cox regression models were used to estimate hazard ratios (HRs) for liver cancer associated with adiposity, stratified by sex and region, and adjusted for age at baseline, education, household income, smoking, alcohol, self‐rated health, and family history of cancer, with age as the time scale and delayed entry at age at baseline. Adiposity measures were modelled both by splitting at sex‐specific quintiles and as continuous variables. WC was modelled using five categories (WC: <70, 70 to <80, 80 to <90, 90 to <100, and ≥ 100 cm), selected to include the International Diabetes Federation (IDF) cut‐off points for central adiposity.^12^ For height‐adjusted weight, standing height was also included in the model as a continuous variable. In further analysis we additionally adjusted for BMI to examine the effects of central adiposity conditionally on general adiposity. To examine which adiposity measure was more strongly associated with risk of liver cancer, we estimated the HR associated with 1‐SD higher adiposity trait. We also compared two models where the first model included all covariates in the main model and the second model included variables in the main model plus each adiposity trait. A likelihood ratio test was used to compare each pair of models. The value of the *χ* ^2^ test statistic and the corresponding *p*‐value were reported for each adiposity trait. When examining adiposity measures as categorical variables, we used ‘floating’ standard errors for the log HR of each adiposity category, so that each HR has a 95% confidence interval (CI) to facilitate comparisons between any two groups.^13^

To adjust for regression dilution bias and obtain the association between usual levels of adiposity and risk of liver cancer, we calculated the correlation between adiposity measures at baseline and first resurvey among 19,788 participants (3 years after baseline). Log HR estimates (and corresponding standard errors) for baseline adiposity measures, which were examined as continuous variables, were divided by this correlation to obtain regression dilution‐adjusted estimates.^14^

We conducted several sensitivity analyses. First, we examined the associations of central adiposity with liver cancer risk in subgroups defined by age, sex, region, education, smoking, alcohol, physical activity, and history of hypertension, diabetes, and cardiovascular disease (CVD). Second, we assessed the HR for liver cancer associated with the number of other baseline metabolic risk factors among participants with central obesity (defined using the IDF categories for Chinese [WC ≥90 cm in men and ≥80 cm in women] or the WHO cut‐off points for substantially increased risk of metabolic complications [WHR ≥ 0.90 in men and ≥0.85 in women]).^15^ Metabolic risk factors were physical inactivity (total physical activity <17.5 metabolic equivalent of task [MET]‐h/day [median]), diabetes (previously diagnosed or screen‐detected), and hypertension (SBP >140 mm Hg and DBP >90 mm Hg or taking anti‐hypertensive medications). Third, we compared the standard and residuals methods to assess the associations of central adiposity (WC, WHR, HC, and WHtR) with liver cancer risk conditionally on BMI. Fourth, we examined the associations of adiposity with mortality from liver cancer. Statistical analysis was done using R version 2.14.2.

## Results

### Baseline characteristics

Among the 478,042 participants included, the mean (SD) baseline age was 51.5 (10.7) years, and 59% were women. Overall, 22.3% of men had WC ≥90 cm and 44.8% of women had WC ≥80 cm. The mean (SD) measured WC at baseline was 82.2 (SD 9.8) cm in men and 79.1 (SD 9.5) cm in women. The mean (SD) WHR was 0.90 (0.06) in men and 0.87 (0.07) in women. There were moderate to strong correlations between measures of general and central adiposity (Supporting Information Table S1). Participants with higher WC were more likely to have lower physical activity and higher SBP and plasma glucose levels (Table 1). They were also more likely to have prevalent diabetes, a history of CVD or hypertension, and a family history of diabetes or cancer. During approximately 5 million person‐years of follow‐up, 1922 participants developed liver cancer at age 30–89 years. 1,049 cases remained after excluding the first 5 years of follow‐up.

**Table 1:** Baseline characteristics of study participants by WC at baseline

	Waist circumference				
Variable	<70	70 to <80	80 to <90	90 to <100	≥100
	(n = 66,063)	(n = 174,202)	(n = 156,514)	(n = 67,073)	(n = 14,190)
Age (SD), year	51.5 (11.2)	51.5 (10.4)	51.6 (10.2)	51.7 (10.5)	51.8 (10.7)
Female, %	71.9	63.8	57.3	47.1	44.9
*Socioeconomic and lifestyle factors*					
Urban region, %	35.2	38.9	48.6	56.6	59.8
≥9 years of education, %	19.8	20.8	21.1	20.0	18.7
Household income ≥35,000 RMB/year, %	13.4	16.6	19.3	20.4	21.4
Ever regular smoking, %					
Male	73.2	70.3	65.2	64.1	64.7
Female	3.5	2.7	2.7	3.1	3.6
Weekly drinking, %					
Male	28.3	33.4	34.0	34.3	34.1
Female	2.0	2.1	2.1	2.2	1.6
Total physical activity (SD), MET‐h/day	22.0 (13.8)	21.8 (14.0)	20.8 (13.6)	19.8 (13.0)	18.4 (12.2)
*Blood pressure and anthropometry*					
SBP (SD), mmHg	123.9 (20.0)	128.2 (20.6)	133.3 (20.6)	138.0 (20.7)	142.8 (21.0)
RPG (SD), mmol/L	5.7 (1.6)	5.9 (1.9)	6.2 (2.4)	6.5 (2.8)	7.0 (3.3)
Body mass index (SD), kg/m^2^	19.5 (1.8)	22.1 (1.9)	24.9 (2.1)	27.8 (2.3)	31.3 (2.9)
Waist circumference (SD), cm	66.2 (3.1)	75.1 (2.8)	84.3 (2.9)	93.6 (2.8)	104.3 (4.5)
Hip circumference (SD), cm	83.6 (4.4)	88.2 (4.4)	93.0 (4.6)	98.1 (5.0)	104.3 (6.3)
Waist‐to‐hip ratio (SD)	0.79 (0.05)	0.85 (0.05)	0.91 (0.05)	0.96 (0.05)	1.00 (0.06)
Percent body fat (SD), %	20.1 (5.8)	25.2 (7.0)	30.5 (7.5)	35.3 (8.0)	40.1 (9.1)
Height (SD), cm	156.8 (7.4)	158.1 (7.7)	159.1 (8.1)	160.3 (8.7)	161.4 (9.1)
*Prior disease history, %*					
Diabetes	2.5	3.9	6.7	9.7	14.2
Coronary heart disease	2.0	2.3	3.2	4.2	4.9
Stroke or TIA	1.0	1.3	1.9	2.4	2.8
Hypertension	4.9	8.1	13.5	19.5	27.5
Family history of diabetes	3.6	4.4	5.4	5.9	6.4
Family history of cancer	13.0	13.6	14.4	14.3	14.6

Means and percentages were adjusted for age, sex, and region (where appropriate).

Abbreviations: MET, metabolic equivalent of task; SBP, systolic blood pressure; RPG, random plasma glucose; TIA, transient ischaemic attack.

### Associations of central adiposity with liver cancer risk

When the IDF cut‐off points were used, the HRs were 1.00 (0.83–1.20), 1.16 (1.04–1.29), 1.20 (1.08–1.33), 1.25 (1.07–1.46), and 1.52 (1.13–2.03) for those with WC <70 cm, 70 to <80 cm, 80 to <90 cm, 90 to <100 cm, and ≥100 cm, respectively (Table 2). The HR per 5 cm higher usual WC was 1.05 (1.01–1.09). WHR was positively associated with liver cancer, with HRs of 1.00 (0.85–1.18), 1.10 (0.95–1.27), 1.07 (0.92–1.25), 1.13 (0.99–1.28), and 1.24 (1.10–1.39) for those in the bottom to the top quintile (Table 2). The HR per 0.1 higher usual WHR was 1.17 (1.03–1.34). Similarly, there was a positive association for WHtR (HR per 0.1: 1.17 [1.01–1.37]) and a positive trend for HC (HR per 5 cm: 1.05 [0.98–1.11], Table 2). When simultaneously adjusting for BMI, the positive associations for WC and WHtR became stronger, while the positive associations for WHR and HC were similar to those from the main model (Table 2). The HR per 5 cm WC was 1.13 (1.04–1.21), and the HR per 0.1 WHtR was 1.49 (1.10–2.03) (Table 2). When the residuals method was used, similar associations were observed (Supporting Information Table 2).

**Table 2:** Standardised incidence rates and adjusted HRs for liver cancer by central adiposity

	No. events	Mean	Incidence	Model 1	Model 2
			per 100,000	HR (95% CI)	HR (95% CI)
*Waist circumference*					
<70	118	66.0	323.7	1.00 (0.83, 1.20)	1.00 (0.77, 1.29)
70 to <80	354	75.0	429.5	1.16 (1.04, 1.29)	1.24 (1.07, 1.45)
80 to <90	356	84.4	447.7	1.20 (1.08, 1.33)	1.40 (1.29, 1.52)
90 to <100	174	93.7	506.8	1.25 (1.07, 1.46)	1.58 (1.30, 1.91)
≥100	47	104.4	475.4	1.52 (1.13, 2.03)	2.10 (1.46, 3.02)
*per 5 cm*				*1.05 (1.01, 1.09)*	*1.13 (1.04, 1.21)*
*per 1‐SD*				*1.09 (1.01, 1.18)*	*1.26 (1.09, 1.46)*
*Waist‐to‐hip ratio*					
Quintile 1	154	0.79	408.7	1.00 (0.85, 1.18)	1.00 (0.83, 1.20)
Quintile 2	193	0.84	451.0	1.10 (0.95, 1.27)	1.11 (0.96, 1.28)
Quintile 3	171	0.88	387.9	1.07 (0.92, 1.25)	1.09 (0.94, 1.26)
Quintile 4	229	0.91	444.9	1.13 (0.99, 1.28)	1.15 (1.01, 1.31)
Quintile 5	302	0.97	495.9	1.24 (1.10, 1.39)	1.28 (1.11, 1.47)
*per 0.1*				*1.17 (1.03, 1.34)*	*1.21 (1.03, 1.42)*
*per 1‐SD*				*1.12 (1.02, 1.23)*	*1.14 (1.02, 1.28)*
*Hip circumference*					
Quintile 1	245	82.0	403.3	1.00 (0.87, 1.15)	1.00 (0.84, 1.20)
Quintile 2	202	87.1	440.1	0.96 (0.84, 1.11)	0.95 (0.82, 1.10)
Quintile 3	203	90.6	428.5	1.05 (0.91, 1.20)	1.01 (0.89, 1.15)
Quintile 4	202	94.2	508.9	1.09 (0.95, 1.25)	1.04 (0.89, 1.20)
Quintile 5	197	100.9	416.9	1.02 (0.87, 1.20)	0.95 (0.77, 1.17)
*per 5 cm*				*1.05 (0.98, 1.11)*	*1.07 (0.97, 1.19)*
*per 1‐SD*				*1.06 (0.98, 1.16)*	*1.10 (0.95, 1.26)*
*Waist‐to‐height ratio*					
Quintile 1	162	0.43	354.5	1.00 (0.85, 1.17)	1.00 (0.81, 1.23)
Quintile 2	197	0.47	452.1	1.18 (1.03, 1.36)	1.24 (1.06, 1.45)
Quintile 3	211	0.50	447.9	1.22 (1.07, 1.40)	1.32 (1.16, 1.50)
Quintile 4	215	0.54	430.9	1.17 (1.02, 1.34)	1.32 (1.14, 1.52)
Quintile 5	264	0.59	480.6	1.29 (1.13, 1.46)	1.54 (1.27, 1.88)
*per 0.1*				*1.17 (1.01, 1.37)*	*1.49 (1.10, 2.03)*
*per 1‐SD*				*1.08 (1.00, 1.17)*	*1.22 (1.05, 1.41)*

Model 1 was stratified by sex and region, and adjusted for age at baseline, education, household income, smoking, alcohol, self‐rated health, and family history of cancer. Model 2 was further adjusted for BMI.

SDs: WC 9.8 cm, WHR 0.07, HC 6.9 cm, WHtR 0.06.

Regression dilution ratios: WC 0.88, WHR 0.70, HC 0.81, WHtR 0.92.

### Comparison across adiposity traits

**Figure 1:**
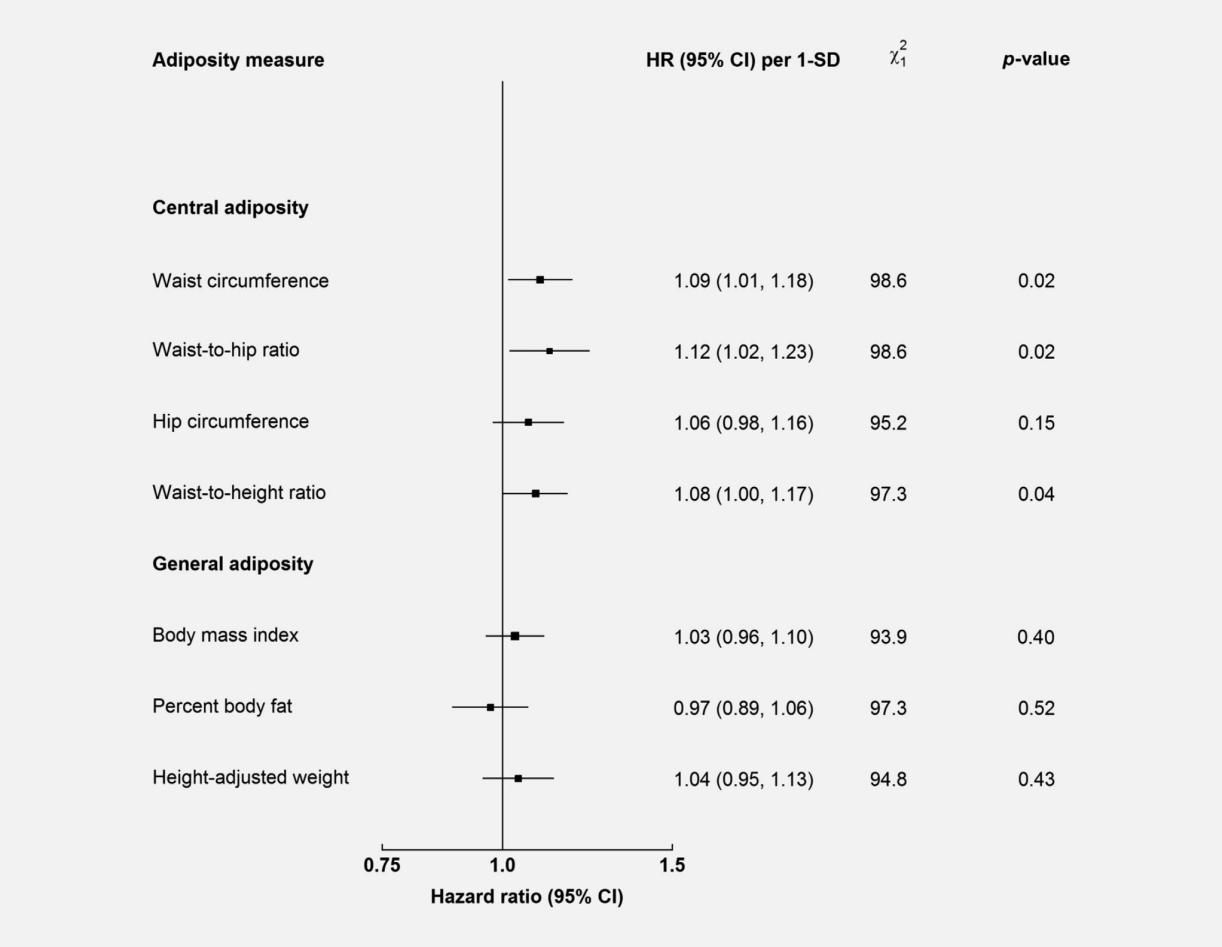
Adjusted HRs for liver cancer by usual levels of central and general adiposity. Model was stratified by sex and region, and adjusted for age at baseline, education, household income, smoking, alcohol, self‐rated health, and family history of cancer. Model 1 included all covariates in the main model. Model 2 included variables in model 1 plus each adiposity trait. The likelihood ratio test was used to compare model 1 and model 2. *χ* ^2^ and *p*‐value from the comparison were reported for each adiposity trait. The sizes of the boxes are proportional to the inverse of the variance of the log hazard ratios.

In contrast to the positive associations for central adiposity, the associations for general adiposity were weaker (Fig. 1 and Supporting Information Table 3). When comparing the HR per 1‐SD increment across measures of central adiposity (Fig. 1), the association was strongest for WHR (1.12 [1.02–1.23]), followed by WC (1.09 [1.01–1.18]), WHtR (1.08 [1.00–1.17]), and HC (1.06 [0.98–1.16]). According to the likelihood ratio test, WHR and WC were the best predictors of liver cancer risk (Fig. 1), with similar c‐statistics (0.584, equal to 3 decimal places).

### Subgroup and sensitivity analyses

**Figure 2:**
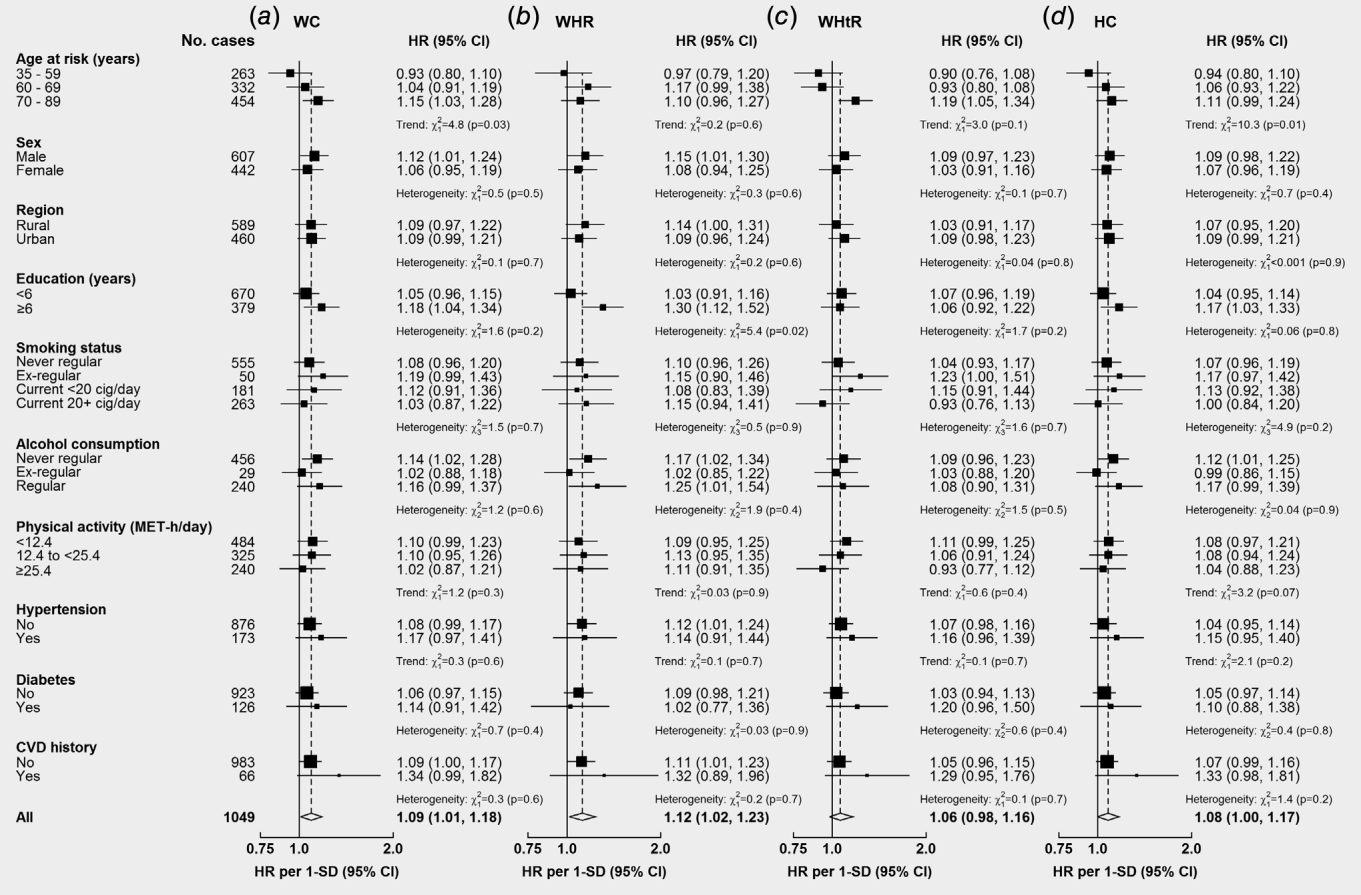
Adjusted HRs for liver cancer by usual levels of central adiposity across population subgroups. Model was stratified by sex and region, and adjusted for age at baseline, education, household income, smoking, alcohol, self‐rated health, and family history of cancer, where appropriate. Boxes represent sex‐specific estimates by subgroup. Diamonds represent the overall HRs. Estimates and 95% CI of the summary HRs are in bold. The sizes of the boxes are proportional to the inverse of the variance of the log hazard ratios. SDs were 9.8 cm for WC, 0.07 for WHR, 6.9 for HC, and 0.06 for WHtR.

**Figure 3:**
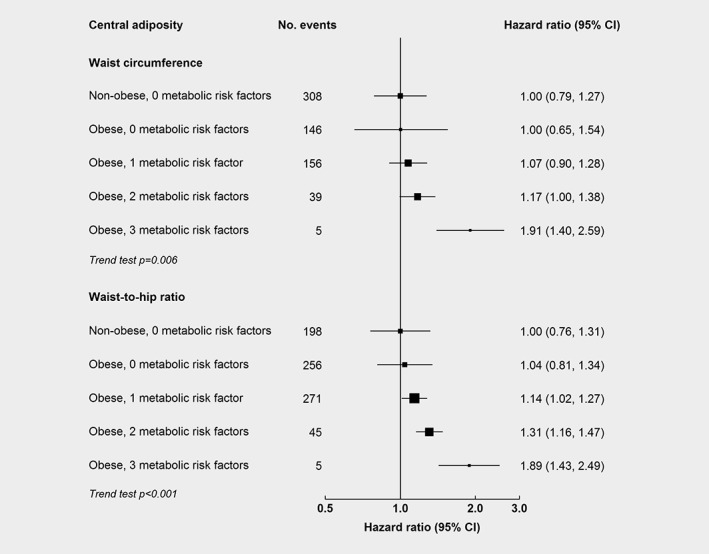
Adjusted HRs for liver cancer by central adiposity by number of metabolic risk factors. Model was stratified by sex and region, and adjusted for age at baseline, education, household income, smoking, alcohol, self‐rated health, and family history of cancer. Central adiposity was defined by WC as ≥90 cm (in men) or ≥ 80 cm (in women) or by WHR as ≥0.90 (in men) or ≥ 0.85 (in women). Metabolic risk factors were physical inactivity (total physical activity <17.5 MET‐h/day [median]), diabetes, and hypertension (SBP >140 mm Hg and DBP >90 mm Hg or taking anti‐hypertensive medications). Boxes represent HRs associated with central adiposity by number of metabolic risk factors. The sizes of the boxes are proportional to the inverse of the variance of the log hazard ratios.

As shown in Figure 2, the associations for central adiposity were stronger at older ages (WC per 1‐SD: 0.93 [0.80–1.10] at 35–59 years, 1.04 [0.91–1.19] at 60–69 years, and 1.15 [1.03–1.26] at 70–89 years, *p* for trend 0.03), but did not differ by region, smoking status, or alcohol consumption. The associations for central adiposity tended to be stronger in men (men: 1.12 [1.01–1.24], women: 1.06 [0.95–1.19]), participants with lower physical activity (<12.4 MET‐h/day: 1.10 [0.99–1.23], 12.4 to <25.4 MET‐h/day: 1.10 [0.95–1.26], ≥25.4 MET‐h/day: 1.02 [0.87–1.21]), and those with hypertension (no: 1.08 [0.99–1.17], yes: 1.17 [0.97–1.41]), diabetes (no: 1.06 [0.97–1.15], yes: 1.14 [0.91–1.42]), and CVD (no: 1.09 [1.00–1.17], yes: 1.34 [0.99–1.82]), but the differences were non‐significant. Compared with individuals without central obesity or metabolic risk factors (physical inactivity, diabetes, and hypertension), the positive associations of central obesity with liver cancer increased progressively with the number of other presenting metabolic risk factors (Fig. 3). The HR for high WC was 1.00 (0.65–1.54), 1.07 (0.90–1.28), 1.17 (1.00–1.38), and 1.91 (1.40–2.59) in those with zero, one, two, and three metabolic risk factors, respectively (*p* for trend 0.006); the corresponding HRs for high WHR were 1.04 (0.81–1.34), 1.14 (1.02–1.27), 1.31 (1.16–1.47), and 1.89 (1.43–2.49) (*p* for trend <0.001). For liver cancer mortality, the associations were similar to those with liver cancer incidence (Supporting Information Table 4). In the analysis without exclusion of the first 5 years of follow‐up, the positive associations for central adiposity attenuated toward the null (Supporting Information Table 5).

## Discussion

In this Chinese population, central adiposity had a positive association with risk of liver cancer, and the association became stronger when adjusting for BMI. In contrast, the positive trend between general adiposity and liver cancer risk was weaker than the positive association for central adiposity. Among central adiposity traits, WHR and WC were the measures most strongly associated with liver cancer risk. Compared with non‐obese and metabolically healthy individuals, the magnitude of the positive association of central adiposity with liver cancer increased progressively with the number of other presenting metabolic risk factors.

Two prospective studies in the United States and Europe have reported positive associations of central adiposity with liver cancer risk.^2^, ^3^ The Liver Cancer Pooling Project in the United States involved 14 prospective cohort studies (2,162 incident liver cancer cases) and reported an 11% higher risk per 5 cm higher WC (HR 1.11 [1.08–1.14]),^3^ while the European Prospective Investigation into Cancer and Nutrition (EPIC), including 177 cases, reported a stronger association for WC (HR per 5 cm 1.25 [1.17–1.33]).^2^ In CKB, the positive association of WC with liver cancer was weaker than the estimates in the U.S. study or EPIC. Likewise, the estimates for WHR, HC, and WHtR in CKB were weaker than those in EPIC.^2^ So far, the only prospective cohort study in East Asia that reported associations of central adiposity with liver cancer risk showed different associations across central adiposity traits.^16^ The Shanghai Women's Health Study with 165 cases reported positive associations for WC and WHtR (HR per 1‐SD: 1.19 [1.02–1.38] *vs*. 1.19 [1.01–1.40]), but no association for WHR (1.01 [0.93–1.09]).^16^ The stronger associations in Western populations may be explained by the different aetiology of liver cancer. While alcohol and NAFLD are major risk factors in Western countries, hepatitis B virus (HBV) is still the most important risk factor in East Asia, particularly in China. NAFLD is associated with risk of liver cancer through the development of cirrhosis, whereas up to 40% of HBV‐related liver cancer is caused by integration of the HBV genome into the host's intracellular DNA.^7^ Moreover, the stronger associations in Western populations may reflect the higher prevalence of metabolic risk factors such as physical inactivity,^17^ which may modify the association of central adiposity with liver cancer risk. In CKB, the positive association of central adiposity with liver cancer increased with the number of other presenting metabolic risk factors (physical inactivity, diabetes, and hypertension). In particular, participants with central adiposity and no metabolic risk factors had similar risk of developing liver cancer to participants without central adiposity and metabolic risk factors.

When additionally adjusting for BMI, the U.S. study reported that the positive association for WC attenuated but remained significant,^3^ while the findings in EPIC were inconsistent across central adiposity traits.^2^ In EPIC, the positive associations for WC and WHR were similar when additionally adjusting for BMI, while the positive association for HC attenuated and became null. In CKB, when adjusting for BMI the positive associations became stronger for all central adiposity traits, particularly for WC and WHtR. Furthermore, we compared the predictive ability of central adiposity measures in relation to liver cancer risk. Although the HRs per 1‐SD were similar for WC, WHR, and WHtR, WHR and WC were the best predictors of liver cancer risk based on both the likelihood ratio test and c‐statistic. Our findings were consistent with a Swedish cohort study which included 259 severe liver disease cases (including 45 of liver cancer) and reported that WHR and WC were more important than other adiposity traits in predicting risk of severe liver disease.^4^

Insulin resistance has been hypothesised as the key mechanism underlying the association between central adiposity and liver cancer risk.^18^, ^19^ Epidemiological studies have shown a positive association of central adiposity (WC or abdominal fat) with insulin resistance assessed by euglycaemic clamp or the insulin sensitivity index, which attenuated toward the null but remained significant when adjusting for BMI.^20^, ^21^ Despite the bi‐directional relationship, insulin resistance is associated with development of NAFLD,^19^ which is a major risk factor for cirrhosis and progression to liver cancer.^6^, ^7^ Indeed, a previous CKB study showed strong positive associations of central adiposity with risk of NAFLD and of NAFLD with risks of cirrhosis and liver cancer.^22^ The same report also showed that WC was more important than BMI in predicting risk of NAFLD.^22^ Furthermore, metabolic risk factors including physical inactivity, hypertension, and diabetes are associated with the insulin resistance syndrome.^18^, ^23^ In CKB, the stronger associations of central obesity with liver cancer risk among participants with increasing numbers of metabolic risk factors lend support to the role of insulin resistance in the aetiology of liver cancer.

The strengths of our study include a prospective study design, a large sample size, a range of adiposity measures, and ability to carefully address confounding (e.g. smoking, alcohol, and HBsAg) and reverse causality (exclusion of early years of follow‐up). However, there were several limitations. First, it is not straightforward to interpret the associations of central adiposity when adjusting for BMI considering the precision of measurement, the functional form of the variables in the model, and the high correlation between these traits (Supporting Information Table S1). However, we showed similar associations using the standard method and the residuals method (Supporting Information Table 2), in line with previous studies that reported consistent estimates among the two methods.^2^, ^16^ Second, although the analyses were restricted to participants without cirrhosis or viral hepatitis at baseline, participants could get HBV infection and develop viral hepatitis during follow‐up. However, the positive associations of central adiposity with liver cancer remained when censoring at diagnosis of viral hepatitis (ICD‐10: B18‐19) during follow‐up (Supporting Information Table 6). Third, as an observational study, our results do not necessarily indicate causality and residual confounding may still exist, especially that related to smoking and alcohol intake. However, the associations of central adiposity with liver cancer risk were not substantially different in never regular smokers or in never regular drinkers (Supporting Information Table 7).

In conclusion, there was a positive association of central adiposity with risk of liver cancer in this Chinese population, which became stronger when adjusting for BMI. WHR and WC had stronger associations with liver cancer risk compared to other central adiposity traits. Our findings suggest that central adiposity, as well as its concomitant metabolic risk factors, might identify individuals at higher risk of liver cancer.

## Supporting information


**Table S1** Correlations between adiposity measures
**Table S2**. Adjusted HRs for liver cancer by central adiposity with additional adjustment for BMI
**Table S3**. Standardised incidence rates and adjusted HRs for liver cancer by general adiposity
**Table S4**. Standardised mortality rates and adjusted HRs for liver cancer by central adiposity
**Table S5**. Adjusted HRs for liver cancer by central adiposity in all participants
**Table S6**. Adjusted HRs for liver cancer associated with central adiposity censoring viral hepatitis
**Table S7**. Adjusted HRs for liver cancer by central adiposity in never regular smokers and never regular drinkers.
