# Supplementary material for: Central adiposity in relation to risk of liver cancer in Chinese adults: A prospective study of 0.5 million people
Source: Int J Cancer. 2019 Feb 13;145(5):1245–53. doi: 10.1002/ijc.32148 (PMC6767784; doi:10.1002/ijc.32148)
Supplement: Supplementary file 1 — Table S1 Correlations between adiposity measures Table S2. Adjusted HRs for liver cancer by central adiposity with additional adjustment for BMI Table S3. Standardised incidence rates and adjusted HRs for liver cancer by general adiposity Table S4. Standardised mortality rates and adjusted HRs for liver cancer by central adiposity Table S5. Adjusted HRs for liver cancer by central adiposity in all participants Table S6. Adjusted HRs for liver cancer associated with central adiposity censoring viral hepatitis Table S7. Adjusted HRs for liver cancer by central adiposity in never regular smokers and never regular drinkers. [file IJC-145-1245-s001.docx]

**Supplementary Material**

**Central adiposity in relation to risk of liver cancer in Chinese adults:**

**a prospective study of 0.5 million people**

**International Journal of Cancer**

Yuanjie Pang^1^, ScM; Christiana Kartsonaki^1,2^, DPhil; Yu Guo^3^, MSc; Yiping Chen^1,2^, DPhil; Ling Yang^1,2^, PhD; Zheng Bian^3^, MSc; Fiona Bragg^1^, DPhil;

Iona Y. Millwood^1,2^, DPhil; Canqing Yu^4^, PhD; Jun Lv^4^, PhD; Junshi Chen^5^, MD;

Liming Li^3,4^, MD; Michael V. Holmes^1,2,6^, PhD; Zhengming Chen^1,2^, DPhil

1. Clinical Trial Service Unit & Epidemiological Studies Unit (CTSU), Nuffield Department of Population Health, University of Oxford, Oxford, UK
2. Medical Research Council Population Health Research Unit (MRC PHRU), Nuffield Department of Population Health, University of Oxford, Oxford, UK
3. Chinese Academy of Medical Sciences, 9 Dongdan San Tiao, Beijing 100730, China
4. School of Public Health, Peking University, Beijing 100191, China
5. National Center for Food Safety Risk Assessment, 37 Guangqu Road, Beijing 100021, China
6. National Institute for Health Research Oxford Biomedical Research Centre, Oxford University Hospital, Old Road, Oxford OX3 7LE, UK

**Address for correspondence**

Dr Christiana Kartsonaki

MRC PHRU, CTSU

Nuffield Department of Population Health

Old Road Campus

University of Oxford

Oxford, OX3 7LF, UK

Fax: 44-1865-743985

Email: christiana.kartsonaki@ndph.ox.ac.uk

Table of Contents

[eMethods 3](#_Toc535331492)

[Supplementary Table 1. Correlations between adiposity measures 7](#_Toc535331493)

[Supplementary Table 2. Adjusted HRs for liver cancer by central adiposity with additional adjustment for BMI 8](#_Toc535331494)

[Supplementary Table 3. Standardised incidence rates and adjusted HRs for liver cancer by general adiposity 9](#_Toc535331495)

[Supplementary Table 4. Standardised mortality rates and adjusted HRs for liver cancer by central adiposity 10](#_Toc535331496)

[Supplementary Table 5. Adjusted HRs for liver cancer by central adiposity in all participants 11](#_Toc535331497)

[Supplementary Table 6. Adjusted HRs for liver cancer associated with central adiposity censoring viral hepatitis 12](#_Toc535331498)

[Supplementary Table 7. Adjusted HRs for liver cancer by central adiposity in never regular smokers and never regular drinkers 13](#_Toc535331499)

# eMethods

*Assessment of smoking and alcohol drinking*

Data collected on smoking included frequency, amount, and type of tobacco smoked, the ages at which participants started smoking regularly and stopped smoking, and the main reason for smoking cessation. Smoking status was classified as never regular, occasional, former regular, or current regular. Never regular smokers were defined as those who reported not smoking at baseline and had smoked <100 cigarettes in lifetime. Former regular smokers (i.e. ex-regular) were defined as those who had smoked a total of at least 100 cigarettes or equivalent but had quit smoking for at least 6 months before baseline by choice. Occasional smokers were defined as those who did not meet the criteria for never smokers or former smoker, and who had not stopped smoking completely for at least the 6 months before baseline. Current regular smokers were defined as those who reported having ever smoked 1 or more cigarettes (or their equivalent) daily for at least 6 months. Among current regular smokers who had stopped 6 or more months before recruitment, approximately half did so because of ill health, and they had more similar risks of diseases to current than former regular smokers.^1,2^ Therefore, they were still counted as current regular smokers in the main analyses. Among current and former regular smokers, information was collected on the types and amount of tobacco smoked when last smoking. Exhaled carbon monoxide was measured to validate smoking status (CareFusion MicroCO meter, CareFusion, Chatham, UK).

Data collected on alcohol drinking included frequency, the ages at which participants began drinking, the type of alcohol (beer, wine or spirits) and the amount of each type consumed on a typical drinking week. Participants were classified into five drinking categories. Abstainers (i.e. never regular) were defined as those who had never or almost never drunk alcohol in the past 12 months and had not drunk weekly in the past. Occasional drinkers were defined as those who in the past 12 months had drunk alcohol occasionally, during certain seasons, or monthly but less than weekly, and had not drunk weekly in the past. Reduced-intake drinkers were those who in the past 12 months had drunk alcohol occasionally, during certain seasons, or monthly but less than weekly, but had drunk weekly in the past. Ex-weekly drinkers (i.e. ex-regular) were those who had drunk weekly in the past but had never or almost never drunk alcohol in the past 12 months. Weekly drinkers (i.e. regular) were those who usually drank at least once a week during the past 12 months.

*Assessment of metabolic risk factors*

Details of physical activity measurements have been described elsewhere.^3^ The physical activity questionnaire was adapted from validated questionnaires (the Shanghai Women’s Health Study^4^ and the European Prospective Investigation into Cancer and Nutrition^5^) and modified after a pilot study. However, these questions had not been validated in CKB separately. The information on usual type and duration of occupational and non-occupational activities (transportation, housework, and leisure-time exercise) during the past year was collected to calculate metabolic equivalent of task (MET) hours per day (MET-h/day), which was the product of the number of hours spent per day participating in each activity and the MET score for that activity. METs from the 2011 update of a major compendium of physical activities were used.^6^

Participants with random plasma glucose (RPG) levels ≥7.8 and <11.1 mmol/L were invited to have a fasting plasma glucose (FPG) test the following day. RPG data were missing in a small number (8156 or 1.6%) of participants, owing to a delay in the availability of on-site test results in certain regions. Previously diagnosed diabetes was defined on the basis of self-reported history of diabetes. Screen-detected diabetes was defined as (i) RPG ≥7.0 mmol/L and time since last eating ≥8 h, or (ii) ≥11.1 mmol/L with time since last eating <8 h, or (iii) a FPG ≥7.0 mmol/L on subsequent testing among participants without previously diagnosed diabetes.^7^

*Statistical analysis*

The models for central adiposity (WC, WHR, and HC) were further adjusted for BMI. Both the standard method and the residuals method were used to examine adjusted associations. In the standard method, BMI was included in the model when examining the adjusted association of WC with liver cancer risk. In the residuals method for central adiposity, the residuals from the regression of each central adiposity trait on BMI were modelled as the main exposure variable and additionally included BMI as a covariate in the model. The residuals were grouped by splitting at quintiles.

References:

1. Chen Z, Peto R, Zhou M, et al. Contrasting male and female trends in tobacco-attributed mortality in China: evidence from successive nationwide prospective cohort studies. *Lancet* 2015;386:1447-1456.

2. Chen ZM, Peto R, Iona A, et al. Emerging tobacco-related cancer risks in China: A nationwide, prospective study of 0.5 million adults. *Cancer* 2015;121 Suppl 17:3097-3106.

3. Du H, Bennett D, Li L, et al. Physical activity and sedentary leisure time and their associations with BMI, waist circumference, and percentage body fat in 0.5 million adults: the China Kadoorie Biobank study. *Am J Clin Nutr* 2013;97:487-496.

4. Matthews CE, Shu XO, Yang G, et al. Reproducibility and validity of the Shanghai Women’s Health Study physical activity questionnaire. *Am J Epidemiol* 2003;158:1114-1122.

5. Wareham NJ, Jakes RW, Rennie KL, et al. Validity and repeatability of the EPIC-Norfolk Physical Activity Questionnaire. *Int J Epidemiol* 2002;31:168-174.

6. Ainsworth BE, Haskell WL, Herrmann SD, et al. 2011 Compendium of Physical Activities: a second update of codes and MET values. *Med Sci Sports Exerc* 2011;43:1575-1581.

7. Bragg F, Li L, Smith M, Guo Y, Chen Y, Millwood I, et al. Associations of blood glucose and prevalent diabetes with risk of cardiovascular disease in 500 000 adult Chinese: the China Kadoorie Biobank. *Diabet Med* 2014;31:540-551.

# Supplementary Table 1. Correlations between adiposity measures

|  |  | **Weight** |  | **BMI** |  | **WC** |  | **HC** |  | **WHR** |  | **WHtR** |  | **BF%** |
| --- | --- | --- | --- | --- | --- | --- | --- | --- | --- | --- | --- | --- | --- | --- |
| **Height** | Men: | 0.53 |  | 0.07 |  | 0.27 |  | 0.42 |  | 0.02 |  | -0.06 |  | 0.04 |
|  | Women: | 0.48 |  | 0.03 |  | 0.2 |  | 0.36 |  | -0.04 |  | -0.12 |  | -0.001 |
| **Weight** |  |  | Men: | 0.88 |  | 0.85 |  | 0.84 |  | 0.54 |  | 0.71 |  | 0.7 |
|  |  |  | Women: | 0.89 |  | 0.83 |  | 0.85 |  | 0.46 |  | 0.68 |  | 0.78 |
| **BMI** |  |  |  |  | Men: | 0.86 |  | 0.76 |  | 0.63 |  | 0.86 |  | 0.8 |
|  |  |  |  |  | Women: | 0.84 |  | 0.78 |  | 0.54 |  | 0.84 |  | 0.89 |
| **WC** |  |  |  |  |  |  | Men: | 0.81 |  | 0.81 |  | 0.95 |  | 0.74 |
|  |  |  |  |  |  |  | Women: | 0.77 |  | 0.79 |  | 0.95 |  | 0.78 |
| **HC** |  |  |  |  |  |  |  |  | Men: | 0.32 |  | 0.7 |  | 0.62 |
|  |  |  |  |  |  |  |  |  | Women: | 0.23 |  | 0.67 |  | 0.7 |
| **WHR** |  |  |  |  |  |  |  |  |  |  | Men: | 0.84 |  | 0.59 |
|  |  |  |  |  |  |  |  |  |  |  | Women: | 0.81 |  | 0.53 |
| **WHtR** |  |  |  |  |  |  |  |  |  |  |  |  | Men: | 0.75 |
|  |  |  |  |  |  |  |  |  |  |  |  |  | Women: | 0.79 |

Partial Pearson correlation coefficients were adjusted for age and region.

# Supplementary Table 2. Adjusted HRs for liver cancer by central adiposity with additional adjustment for BMI

|  | **Basic** | **Residuals** |
| --- | --- | --- |
|  | **HR (95% CI)** | **HR (95% CI)** |
| **Waist circumference** | |  |
| Quintile 1 | 1.00 (0.86, 1.16) | 1.00 (0.84, 1.19) |
| Quintile 2 | 1.03 (0.89, 1.19) | 1.05 (0.90, 1.23) |
| Quintile 3 | 1.11 (0.97, 1.27) | 1.09 (0.95, 1.25) |
| Quintile 4 | 1.10 (0.96, 1.26) | 1.15 (1.01, 1.31) |
| Quintile 5 | 1.23 (1.08, 1.41) | 1.39 (1.23, 1.58) |
|  |  |  |
| **Waist-to-hip ratio** |  |  |
| Quintile 1 | 1.00 (0.83, 1.20) | 1.00 (0.83, 1.21) |
| Quintile 2 | 1.11 (0.96, 1.28) | 0.90 (0.77, 1.06) |
| Quintile 3 | 1.09 (0.94, 1.26) | 1.05 (0.92, 1.20) |
| Quintile 4 | 1.15 (1.01, 1.31) | 0.97 (0.85, 1.10) |
| Quintile 5 | 1.28 (1.11, 1.47) | 1.16 (1.03, 1.31) |
|  |  |  |
| **Hip circumference** | |  |
| Quintile 1 | 1.00 (0.84, 1.20) | 1.00 (0.87, 1.15) |
| Quintile 2 | 0.95 (0.82, 1.10) | 1.02 (0.89, 1.16) |
| Quintile 3 | 1.01 (0.89, 1.15) | 0.93 (0.80, 1.07) |
| Quintile 4 | 1.04 (0.89, 1.20) | 1.04 (0.90, 1.20) |
| Quintile 5 | 0.95 (0.77, 1.17) | 1.13 (0.97, 1.33) |
|  |  |  |
| **Waist-to-height ratio** | |  |
| Quintile 1 | 1.00 (0.81, 1.23) | 1.00 (0.84, 1.19) |
| Quintile 2 | 1.24 (1.06, 1.45) | 1.12 (0.96, 1.30) |
| Quintile 3 | 1.32 (1.16, 1.50) | 1.16 (1.01, 1.33) |
| Quintile 4 | 1.32 (1.14, 1.52) | 1.35 (1.20, 1.53) |
| Quintile 5 | 1.54 (1.27, 1.88) | 1.39 (1.22, 1.59) |

Model was stratified by sex and region, and adjusted for age at baseline, education, household income,

smoking, alcohol, self-rated health, family history of cancer, and BMI.

# Supplementary Table 3. Standardised incidence rates and adjusted HRs for liver cancer by general adiposity

|  | **No. events** | **Mean** | **Incidence** | **HR (95% CI)** |
| --- | --- | --- | --- | --- |
|  |  |  | **per 100,000** |  |
| **BMI** |  |  |  |  |
| <18.5 | 50 | 17.5 | 407.1 | 1.00 (0.75, 1.33) |
| 18.5 to <22.5 | 357 | 20.8 | 448.6 | 1.05 (0.94, 1.17) |
| 22.5 to <25.0 | 295 | 23.7 | 443.5 | 1.09 (0.98, 1.23) |
| 25.0 to <27.5 | 198 | 26.1 | 408.0 | 1.04 (0.90, 1.20) |
| 27.5 to <30.0 | 99 | 28.5 | 500.7 | 1.16 (0.95, 1.41) |
| ≥30.0 | 50 | 31.9 | 497.0 | 1.35 (1.02, 1.79) |
| *per 5 units* |  |  |  | *1.04 (0.95, 1.15)* |
| *Per 1-SD* |  |  |  | *1.03 (0.96, 1.10)* |
|  |  |  |  |  |
| **Percent body fat** | |  |  |  |
| Quintile 1 | 236 | 19.0 | 460.1 | 1.00 (0.87, 1.14) |
| Quintile 2 | 226 | 24.1 | 451.7 | 1.03 (0.90, 1.17) |
| Quintile 3 | 195 | 27.7 | 439.8 | 0.89 (0.77, 1.02) |
| Quintile 4 | 196 | 31.3 | 420.8 | 0.91 (0.79, 1.05) |
| Quintile 5 | 196 | 37.8 | 423.3 | 0.92 (0.80, 1.07) |
| *per 5%* |  |  |  | *0.98 (0.93, 1.04)* |
| *per 1-SD* |  |  |  | *0.97 (0.89, 1.06)* |
|  |  |  |  |  |
| **Height-adjusted weight** | |  |  |  |
| Quintile 1 | 249 | 46.7 | 423.7 | 1.00 (0.86, 1.16) |
| Quintile 2 | 224 | 54.0 | 471.7 | 1.06 (0.93, 1.21) |
| Quintile 3 | 193 | 59.0 | 445.0 | 0.97 (0.84, 1.11) |
| Quintile 4 | 180 | 64.5 | 436.7 | 0.94 (0.81, 1.09) |
| Quintile 5 | 203 | 74.7 | 478.0 | 1.13 (0.96, 1.33) |
| *per 5 kg* |  |  |  | *1.02 (0.98, 1.06)* |
| *per 1-SD* |  |  |  | *1.04 (0.95, 1.13)* |

Model was stratified by sex and region, and adjusted for age at baseline, education, household income, smoking, alcohol, self-rated health, and family history of cancer.

SDs: BMI 3.4 kg/m^2^, %BF 8.4%, weight 10.8 kg.

Regression dilution ratios: BMI 0.95, %BF 0.88, weight 0.96.

# Supplementary Table 4. Standardised mortality rates and adjusted HRs for liver cancer by central adiposity

|  | **No. events** | **Mortality** | **HR (95% CI)** |
| --- | --- | --- | --- |
|  |  | **per 100,000** |  |
| **Waist circumference** | |  |  |
| Quintile 1 | 75 | 224.4 | 1.00 (0.79, 1.26) |
| Quintile 2 | 256 | 314.2 | 1.30 (1.15, 1.48) |
| Quintile 3 | 229 | 288.3 | 1.20 (1.06, 1.37) |
| Quintile 4 | 121 | 338.5 | 1.35 (1.12, 1.62) |
| Quintile 5 | 31 | 189.4 | 1.53 (1.07, 2.20) |
| *per 5 cm* |  |  | *1.04 (1.00, 1.09)* |
| *per 1-SD* |  |  | *1.09 (0.99, 1.19)* |
|  |  |  |  |
| **Waist-to-hip ratio** | |  |  |
| Quintile 1 | 108 | 282.1 | 1.00 (0.82, 1.21) |
| Quintile 2 | 130 | 291.6 | 1.05 (0.88, 1.25) |
| Quintile 3 | 121 | 299.2 | 1.07 (0.90, 1.28) |
| Quintile 4 | 153 | 304.5 | 1.06 (0.91, 1.25) |
| Quintile 5 | 200 | 314.8 | 1.14 (0.99, 1.32) |
| *per 0.1* |  |  | *1.12 (0.95, 1.31)* |
| *per 1-SD* |  |  | *1.08 (0.96, 1.21)* |
|  |  |  |  |
| **Hip circumference** | |  |  |
| Quintile 1 | 159 | 257.8 | 1.00 (0.85, 1.18) |
| Quintile 2 | 140 | 296.3 | 1.03 (0.87, 1.22) |
| Quintile 3 | 144 | 300.7 | 1.16 (0.98, 1.36) |
| Quintile 4 | 138 | 346.4 | 1.17 (0.99, 1.38) |
| Quintile 5 | 131 | 255.6 | 1.09 (0.90, 1.32) |
| *per 5 cm* |  |  | *1.06 (0.99, 1.15)* |
| *per 1-SD* |  |  | *1.09 (0.98, 1.21)* |
|  |  |  |  |
| **Waist-to-height ratio** | |  |  |
| Quintile 1 | 105 | 246.3 | 1.00 (0.82, 1.22) |
| Quintile 2 | 149 | 346.7 | 1.38 (1.17, 1.62) |
| Quintile 3 | 131 | 288.6 | 1.17 (0.99, 1.39) |
| Quintile 4 | 146 | 295.9 | 1.23 (1.04, 1.44) |
| Quintile 5 | 181 | 294.6 | 1.35 (1.15, 1.57) |
| *per 0.1* |  |  | *1.19 (0.99, 1.44)* |
| *per 1-SD* |  |  | *1.09 (0.99, 1.19)* |

Model 1 was stratified by sex and region, and adjusted for age at baseline, education, household income, smoking, alcohol, self-rated health, and family history of cancer. Model 2 was further adjusted for BMI.

SDs: WC 9.8 cm, WHR 0.07, HC 6.9 cm, WHtR 0.06.

Regression dilution ratios: WC 0.88, WHR 0.70, HC 0.81, WHtR 0.92.

# Supplementary Table 5. Adjusted HRs for liver cancer by central adiposity in all participants

|  | **No. events** | **Model 1** | **Model 2** |
| --- | --- | --- | --- |
|  |  | **HR (95% CI)** | **HR (95% CI)** |
| **Waist circumference** | |  |  |
| Quintile 1 | 253 | 1.00 (0.88, 1.14) | 1.00 (0.84, 1.20) |
| Quintile 2 | 670 | 1.04 (0.96, 1.12) | 1.18 (1.06, 1.32) |
| Quintile 3 | 622 | 1.00 (0.93, 1.08) | 1.31 (1.23, 1.40) |
| Quintile 4 | 302 | 1.02 (0.91, 1.14) | 1.53 (1.32, 1.77) |
| Quintile 5 | 75 | 1.10 (0.87, 1.39) | 1.96 (1.47, 2.60) |
| *per 5 cm* |  | *1.00 (0.97, 1.03)* | *1.10 (1.04, 1.16)* |
| *per 1-SD* |  | *0.99 (0.94, 1.05)* | *1.20 (1.07, 1.33)* |
|  |  |  |  |
| **Waist-to-hip ratio** |  |  |  |
| Quintile 1 | 308 | 1.00 (0.89, 1.12) | 1.00 (0.88, 1.14) |
| Quintile 2 | 354 | 1.03 (0.93, 1.14) | 1.08 (0.97, 1.21) |
| Quintile 3 | 321 | 1.04 (0.93, 1.16) | 1.13 (1.01, 1.26) |
| Quintile 4 | 419 | 1.06 (0.97, 1.17) | 1.20 (1.09, 1.33) |
| Quintile 5 | 520 | 1.09 (1.00, 1.19) | 1.30 (1.17, 1.45) |
| *per 0.1* |  | *1.06 (0.96, 1.17)* | *1.21 (1.08, 1.37)* |
| *per 1-SD* |  | *1.04 (0.97, 1.12)* | *1.15 (1.05, 1.25)* |
|  |  |  |  |
| **Hip circumference** | |  |  |
| Quintile 1 | 508 | 1.00 (0.91, 1.10) | 1.00 (0.88, 1.14) |
| Quintile 2 | 382 | 0.88 (0.79, 0.97) | 0.90 (0.81, 1.00) |
| Quintile 3 | 357 | 0.89 (0.80, 0.99) | 0.93 (0.84, 1.03) |
| Quintile 4 | 335 | 0.87 (0.78, 0.97) | 0.93 (0.83, 1.05) |
| Quintile 5 | 340 | 0.85 (0.76, 0.96) | 0.94 (0.80, 1.11) |
| *per 5 cm* |  | *0.96 (0.92, 1.01)* | *1.01 (0.94, 1.09)* |
| *per 1-SD* |  | *0.95 (0.89, 1.01)* | *1.01 (0.91, 1.13)* |
|  |  |  |  |
| **Waist-to-height ratio** | |  |  |
| Quintile 1 | 356 | 1.00 (0.90, 1.11) | 1.00 (0.87, 1.16) |
| Quintile 2 | 374 | 1.04 (0.94, 1.15) | 1.12 (1.00, 1.25) |
| Quintile 3 | 376 | 1.02 (0.92, 1.12) | 1.15 (1.05, 1.27) |
| Quintile 4 | 372 | 0.95 (0.86, 1.05) | 1.14 (1.02, 1.27) |
| Quintile 5 | 444 | 0.99 (0.90, 1.09) | 1.31 (1.13, 1.53) |
| *per 0.1* |  | *0.96 (0.86, 1.08)* | *1.34 (1.06, 1.68)* |
| *per 1-SD* |  | *0.98 (0.93, 1.04)* | *1.15 (1.03, 1.29)* |

Model 1 was stratified by sex and region, and adjusted for age at baseline, education, household income, smoking, alcohol, self-rated health, and family history of cancer. Model 2 was further adjusted for BMI.

SDs: WC 9.8 cm, WHR 0.07, HC 6.9 cm, WHtR 0.06.

Regression dilution ratios: WC 0.88, WHR 0.70, HC 0.81, WHtR 0.92.

# Supplementary Table 6. Adjusted HRs for liver cancer associated with central adiposity censoring viral hepatitis

|  | | **All participants** | |  | **Censoring viral hepatitis** | |
| --- | --- | --- | --- | --- | --- | --- |
|  | | **No. cases** | **HR (95% CI)** |  | **No. cases** | **HR (95% CI)** |
| **Waist circumference** | | |  |  |  |  |
| <70 | | 118 | 1.00 (0.83, 1.20) |  | 114 | 1.00 (0.83, 1.21) |
| 70 to <80 | | 354 | 1.16 (1.04, 1.29) |  | 330 | 1.12 (1.01, 1.26) |
| 80 to <90 | | 356 | 1.20 (1.08, 1.33) |  | 341 | 1.20 (1.08, 1.33) |
| 90 to <100 | | 174 | 1.25 (1.07, 1.46) |  | 172 | 1.29 (1.10, 1.50) |
| ≥100 | | 47 | 1.52 (1.13, 2.03) |  | 46 | 1.55 (1.15, 2.08) |
| *per 5 cm* | |  | *1.05 (1.01, 1.09)* |  |  | *1.05 (1.01, 1.10)* |
| *per 1-SD* | |  | *1.09 (1.01, 1.18)* |  |  | *1.11 (1.03, 1.20)* |
|  | |  |  |  |  |  |
| **Waist-to-hip ratio** | | |  |  |  |  |
| Quintile 1 | | 154 | 1.00 (0.85, 1.18) |  | 146 | 1.00 (0.85, 1.18) |
| Quintile 2 | | 193 | 1.10 (0.95, 1.27) |  | 183 | 1.11 (0.96, 1.28) |
| Quintile 3 | | 171 | 1.07 (0.92, 1.25) |  | 161 | 1.07 (0.92, 1.25) |
| Quintile 4 | | 229 | 1.13 (0.99, 1.28) |  | 224 | 1.17 (1.02, 1.33) |
| Quintile 5 | | 302 | 1.24 (1.10, 1.39) |  | 289 | 1.26 (1.12, 1.42) |
| *per 0.1* | |  | *1.17 (1.03, 1.34)* |  |  | *1.19 (1.04, 1.36)* |
| *per 1-SD* | | | *1.12 (1.02, 1.23)* |  |  | *1.13 (1.03, 1.24)* |
|  | |  |  |  |  |  |
| **Hip circumference** | | |  |  |  |  |
| Quintile 1 | | 245 | 1.00 (0.87, 1.15) |  | 231 | 1.00 (0.87, 1.15) |
| Quintile 2 | | 202 | 0.96 (0.84, 1.11) |  | 197 | 1.00 (0.87, 1.15) |
| Quintile 3 | | 203 | 1.05 (0.91, 1.20) |  | 188 | 1.03 (0.90, 1.19) |
| Quintile 4 | | 202 | 1.09 (0.95, 1.25) |  | 193 | 1.11 (0.96, 1.28) |
| Quintile 5 | | 197 | 1.02 (0.87, 1.20) |  | 194 | 1.07 (0.91, 1.26) |
| *per 5 cm* | | | *1.05 (0.98, 1.11)* |  |  | *1.06 (0.99, 1.12)* |
| *per 1-SD* | |  | *1.06 (0.98, 1.16)* |  |  | *1.08 (0.99, 1.18)* |
|  | |  |  |  |  |  |
| **Waist-to-height ratio** | | |  |  |  |  |
| Quintile 1 | | 162 | 1.00 (0.85, 1.17) |  | 156 | 1.00 (0.85, 1.18) |
| Quintile 2 | | 197 | 1.18 (1.03, 1.36) |  | 182 | 1.14 (0.99, 1.32) |
| Quintile 3 | | 211 | 1.22 (1.07, 1.40) |  | 200 | 1.21 (1.05, 1.38) |
| Quintile 4 | | 215 | 1.17 (1.02, 1.34) |  | 205 | 1.16 (1.01, 1.33) |
| Quintile 5 | 264 | | 1.29 (1.13, 1.46) |  | 260 | 1.32 (1.16, 1.50) |
| *per 0.1* | |  | *1.17 (1.01, 1.37)* |  |  | *1.22 (1.04, 1.42)* |
| *per 1-SD* | |  | *1.08 (1.00, 1.17)* |  |  | *1.10 (1.02, 1.19)* |

Model was stratified by sex and region, and adjusted for age at baseline, education, household income, smoking, alcohol, self-rated health, and family history of cancer.

SDs: WC 9.8 cm, WHR 0.07, HC 6.9 cm, WHtR 0.06.

Regression dilution ratios: WC 0.88, WHR 0.70, HC 0.81, WHtR 0.92.

# Supplementary Table 7. Adjusted HRs for liver cancer by central adiposity in never regular smokers and never regular drinkers

|  | **Never regular smokers** | |  | **Never regular drinkers** | |
| --- | --- | --- | --- | --- | --- |
|  | **No. events** | **HR (95% CI)** |  | **No. events** | **HR (95% CI)** |
| **Waist circumference** | |  |  |  |  |
| <70 | 74 | 1.00 (0.79, 1.26) |  | 156 | 1.06 (0.90, 1.24) |
| 70 to <80 | 174 | 0.98 (0.84, 1.14) |  | 67 | 1.00 (0.78, 1.28) |
| 80 to <90 | 193 | 1.11 (0.96, 1.27) |  | 138 | 1.05 (0.89, 1.24) |
| 90 to <100 | 93 | 1.22 (0.99, 1.50) |  | 78 | 1.43 (1.14, 1.80) |
| ≥100 | 21 | 1.26 (0.81, 1.95) |  | 17 | 1.45 (0.89, 2.35) |
| *per 5 cm* |  | *1.06 (1.01, 1.12)* |  |  | *1.07 (1.01, 1.13)* |
| *per 1-SD* |  | *1.12 (1.01, 1.24)* |  |  | *1.14 (1.02, 1.28)* |
|  |  |  |  |  |  |
| **Waist-to-hip ratio** | |  |  |  |  |
| Quintile 1 | 74 | 1.00 (0.79, 1.26) |  | 68 | 0.84 (0.66, 1.06) |
| Quintile 2 | 92 | 1.06 (0.86, 1.30) |  | 77 | 1.00 (0.79, 1.26) |
| Quintile 3 | 97 | 1.20 (0.98, 1.46) |  | 71 | 0.98 (0.78, 1.23) |
| Quintile 4 | 116 | 1.07 (0.89, 1.29) |  | 96 | 1.01 (0.83, 1.24) |
| Quintile 5 | 176 | 1.27 (1.09, 1.48) |  | 144 | 1.18 (0.99, 1.40) |
| *per 0.1* |  | *1.21 (1.02, 1.45)* |  |  | *1.25 (1.03, 1.51)* |
| *per 1-SD* |  | *1.15 (1.01, 1.30)* |  |  | *1.17 (1.02, 1.34)* |
|  |  |  |  |  |  |
| **Hip circumference** | |  |  |  |  |
| Quintile 1 | 125 | 1.00 (0.83, 1.21) |  | 93 | 0.94 (0.77, 1.15) |
| Quintile 2 | 89 | 0.82 (0.66, 1.01) |  | 116 | 1.00 (0.82, 1.21) |
| Quintile 3 | 119 | 1.14 (0.95, 1.36) |  | 88 | 1.00 (0.82, 1.24) |
| Quintile 4 | 109 | 1.07 (0.89, 1.29) |  | 78 | 0.97 (0.78, 1.22) |
| Quintile 5 | 113 | 1.02 (0.83, 1.25) |  | 81 | 1.03 (0.81, 1.31) |
| *per 5 cm* |  | *1.06 (0.98, 1.15)* |  |  | *1.06 (0.97, 1.16)* |
| *per 1-SD* |  | *1.08 (0.97, 1.21)* |  |  | *1.09 (0.96, 1.23)* |
|  |  |  |  |  |  |
| **Waist-to-height ratio** | |  |  |  |  |
| Quintile 1 | 76 | 1.00 (0.79, 1.26) |  | 82 | 1.12 (0.90, 1.39) |
| Quintile 2 | 93 | 1.14 (0.93, 1.40) |  | 76 | 1.00 (0.79, 1.26) |
| Quintile 3 | 102 | 1.11 (0.91, 1.35) |  | 82 | 1.07 (0.86, 1.33) |
| Quintile 4 | 125 | 1.22 (1.02, 1.45) |  | 79 | 0.96 (0.77, 1.20) |
| Quintile 5 | 159 | 1.34 (1.13, 1.57) |  | 137 | 1.47 (1.23, 1.75) |
| *per 0.1* |  | *1.25 (1.02, 1.54)* |  |  | *1.27 (1.02, 1.59)* |
| *per 1-SD* |  | *1.12 (1.01, 1.23)* |  |  | *1.12 (1.01, 1.25)* |

Model was stratified by sex and region, and adjusted for age at baseline, education, household income, smoking, alcohol, self-rated health, and family history of cancer, where appropriate.

SDs: WC 9.8 cm, WHR 0.07, HC 6.9 cm, WHtR 0.06.

Regression dilution ratios: WC 0.88, WHR 0.70, HC 0.81, WHtR 0.92.

Never regular drinkers included never regular, ex-regular, and regular drinkers.
